# The impact of GPX1 on the association of groundwater selenium and depression: a project FRONTIER study

**DOI:** 10.1186/1471-244X-13-7

**Published:** 2013-01-04

**Authors:** Leigh A Johnson, Jack A Phillips, Cortney Mauer, Melissa Edwards, Valerie Hobson Balldin, James R Hall, Robert Barber, Tori L Conger, Eric J Ho, Sid E O’Bryant

**Affiliations:** 1 Department of Internal Medicine, Institute for Aging & Alzheimer’s Disease Research, University of North Texas Health Science Center, 3500 Camp Bowie Blvd, Fort Worth, TX, 76107, USA; 2 Department of Psychology, Texas Tech University, Lubbock, TX, USA; 3 Department of Psychology, University of North Texas, Denton, TX, USA; 4 Department of Internal Medicine, University of North Texas Health, Science Center, Fort Worth, TX, USA; 5 Department of Psychiatry, University of North Texas Health Science Center, Fort Worth, TX, USA; 6 Department of Pharmacology & Neuroscience, Institute for Aging & Alzheimer’s Disease Research, University of North Texas Health Science Center, Fort Worth, TX, USA; 7 Department of Internal Medicine, University of North Texas Health Science Center, Fort Worth, TX, USA; 8 Texas College of Osteopathic Medicine, University of North Texas Health Science Center, Fort Worth, TX, USA; 9 Department of Internal Medicine, Institute for Aging & Alzheimer’s Disease Research, University of North Texas Health Science Center, Fort Worth, TX, USA

**Keywords:** Aging, Depression, Environmental factors, Selenium, GPX1

## Abstract

**Background:**

Prior animal model and human-based studies have linked selenium concentrations to decreased risk for depression; however, this work has not focused on household groundwater levels or specific depressive symptoms. The current study evaluated the link between groundwater selenium levels and depression. We also sought to determine if a functional polymorphism in the glutathione peroxidase 1 (GPX1) gene impacted this link.

**Methods:**

We used a cross-sectional design to analyze data from 585 participants (183 men and 402 women) from Project FRONTIER, a study of rural health in West Texas. Residential selenium concentrations were estimated using Geospatial Information System (GIS) analyses. Linear regression models were created using Geriatric Depression Scale (GDS-30) total and subfactor scores as outcome variables and selenium concentrations as predictor variables. Analyses were re-run after stratification of the sample on GPX1 Pro198Leu genotype (rs1050454).

**Results:**

Selenium levels were significantly and negatively related to all GDS and subfactor scores accounting for up to 17% of the variance beyond covariates. Selenium was most strongly protective against depression among homozygous carriers of the C allele at the Pro198Leu polymorphism of the GPX1 gene. Analyses also point towards a gene-environmental interaction between selenium exposure and GPX1 polymorphism.

**Conclusion:**

Our results support the link between groundwater selenium levels and decreased depression symptoms. These findings also highlight the need to consider the genetics of the glutathione peroxidase system when examining this relationship, as variation in the GPX1 gene is related to depression risk and significantly influences the protective impact of selenium, which is indicative of a gene-environment interaction.

## Background

Depression is the most frequently occurring psychiatric disorder [1] with 1 in 10 adults reporting depression [2]. Depression has been shown to have a wide variety of adverse health outcomes including poorer physical and social functioning, and decreased quality of life [3-5] as well as an increase in morbidity and mortality [6-8]. Understanding the link between depression and environmental factors that may influence its occurrence, symptom presentation, and severity, may have a significant impact on our understanding of the biological mechanisms underlying depression and its treatment. Past research has shown relationships between depression and environmental factors, such as natural sour gas containing H_2_S [9] and pesticides [10]. Another naturally occurring substance that has been related to depression is selenium.

Past research has shown that selenoproteins are essential for numerous biological functions [11], many of which are thought to be related to depression. Depression is thought to be unpinned by multiple pathways, such as inflammation, oxidative and nitrosative stress, as well as a decrease in antioxidant levels [12-14]. Selenium, in the form of Selenoprotein P, protects the brain against oxidative damage [15] and is involved in the regulation of the inflammatory response [11]. Additionally selenium, an antioxidant and an essential component in the glutathione peroxidase system, protects lipids in polyunsaturated membranes from oxidative degradation. Depression is often associated with lower levels of antioxidants, such as glutathione and GPx, glutathione peroxidase enzyme [16,17]. These findings suggest that selenium may be protective against depression, and selenium supplementation may have the potential to alleviate the symptoms of depression. However, research investigating the relationship between selenium and depression has yielded inconsistent findings. Low selenium concentrations have been related to increased incidence of many human diseases including depression [18-20]. Rayman et al. [21] failed to identify any benefit in mood or quality of life in a sample of elderly participants. Prenatal selenium supplementation has been linked to increased serum selenium levels as well as decreased risk for postpartum depression [22]. Pasco et al. [23] found that lower dietary selenium was related to a new diagnosis of major depression. Recently, Gao et al. [24] found that higher selenium were associated with lower GDS scores; however this effect was no longer significant when cognitive functioning was added to the model. This finding is interesting because it suggests that the link between selenium and depression may be impacted by individual factors, such as genetic variants that may impact the selenium levels in the body.

Selenium also plays a key role in proper functioning of the glutathione peroxidase system, which has been linked to depression. The glutathione peroxidase enzyme (GPx) is expressed in lower amounts among depressed patients, and GPx activity has been linked to depressed mood and autonomic symptoms [25]. Because functional variants have been identified within glutathione peroxidase genes, genetic variation within the glutathione peroxidase pathway is an important consideration when examining selenium-health status links. In particular, the Pro198Leu polymorphism of the glutathione peroxidase 1 (GPX1) gene (rs1050454) has received a great deal of attention recently in the literature [26]. This polymorphism contains a c.599C>T SNP, which is also referred to as rs1050450. The single nucleotide polymorphism (SNP) likely affects the activity of variant proteins [27]. The T allele of the rs1050450 GPX1 SNP has been associated with bladder, lung, and breast cancers, intracerebral hemorrhage [28], metabolic syndrome [29], and coronary artery disease [30]. It is functional in humans and impacts the response of GPX1 activity to selenium [31]. Genetic polymorphisms of the GPX1 gene have been shown to be related to tumor susceptibility [32], blood selenium levels associated with supplementation [33], and other health conditions. However, to date, we are aware of no studies that have examined the impact of GPX1 polymorphisms on the selenium- depression link.

The current study was designed to (a) study the link between groundwater selenium levels and depressive symptoms, (b) determine if the Pro198Leu GPX1 directly impact depression risk, and (c) determine if the Pro198Leu polymorphisms of the GPX1 gene impact these findings. It was hypothesized that higher groundwater selenium would be protective against depressive symptoms. No specific a priori hypotheses were made regarding the Pro198Leu polymorphism given that this is the first study to examine the role of this genetic variant in depression.

## Methods

### Participants and procedures

Data were analyzed from 585 participants (183 men and 402 women) from Project FRONTIER. Project FRONTIER is an ongoing study of factors impacting rural health that utilizes a community-based participatory research (CBPR) approach. The CBPR approach of partnering with community members and organizations is particularly useful when working with underserved communities, thus it is very well suited to rural health research and has been recommended by the National Institute on Environmental Health Sciences [34]. All participants signed written informed consents. Once participants have completed the consent process, s/he undergoes an interview (participant and informant), a standardized medical examination, clinical labs, and neuropsychological testing. Inclusion criteria are (a) age 40 and above and (b) residing in one of the counties included in Project FRONTIER. The counties currently included in project FRONTIER are Cochran County, Bailey County, and Parmer County, all of which are located on the Texas – New Mexico border. Participant recruitment is completed by community recruiters/assessors through multiple means including brochures/flyers, presentations and events, in-person and/or door-to-door solicitation, as well as snowball recruitment. Our prior work has shown that the research sample closely matches the demographics of the community at large [34]. Project FRONTIER is conducted under an IRB approved protocol. We have published findings utilizing this approach with Project FRONTIER elsewhere [35-37]. Project FRONTIER is conducted under the approval of Texas Tech Health Sciences Center IRB Board, L06-028. This archival analysis was conducted from a de-identified database under the approval of the University of North Texas Health Sciences Center IRB Review Board, protocol number 2012-071.

### Measures

#### Geriatric depression scale (GDS-30)

The Geriatric Depression Scale was created specifically for screening depressive symptoms among older populations [38], and has been widely utilized in both clinical and research settings. The GDS has demonstrated adequate internal consistency, test–retest reliability, and concurrent validity [39]. A recent factor analytic study [40] identified a four- factor solution underlying the scale. The Dysphoria factor contains 11 items primarily associated with a sad mood; the Meaninglessness factor consists of seven items that reflect an appraisal of the meaning (or lack thereof) in one’s life; the Apathy factor is made up of six items that reflect a lack of motivation or initiative; finally, the Cognitive Impairment factor consists of six items that reflect difficulty and concern with cognitive processes.

#### Determination of GIS-selenium

Geographic information system (GIS) analysis is a way of displaying and analyzing geographically referenced information that is frequently used to estimate environmental exposures [35,41-46]. We used the Environmental Systems Research Institute ArcGIS (release 9.2) program to plot a point for each of the selenium ground water measurements from the Texas Water Development Board (TWDB). Through inverse distance weighted (IDW) interpolation, the ArcGIS software built a three-dimensional surface map from a list of points. Each point’s influence was weighted based off its distance to that section of the map, which was generated using 12 well measurements from the TWDB within the immediate geographic vicinity. Each of the study participant’s current residential address was geocoded with the ArcGIS StreetMap data. Finally, GIS-selenium concentration was calculated by extracting the estimated selenium value from the IDW surface at each resident’s location. The inter-rater reliability of our GIS approach to calculating our groundwater elements is very high (Pearson Correlation coefficient > 0.9). In our prior work, we have found our GIS-based estimates to be very similar to concentrations found from direct measurement [35].

Polymerase chain reactions (PCRs) were utilized to genotype the GPX1 (rs1050450) polymorphism. Primers were purchased from Integrated DNA Technologies, Inc.: FW 5^′^- CTACGCAGGTACAGC-CGCCGCT-3^′^ and RV 5^′^-AAGGTGTTCCTCCCTCG-TAGGT-3^′^. Amplification conditions included: 100ng template DNA, DMSO, water, and GoTaq Green Master Mix (3mM MgCl_2_, 1X PCR buffer, 400 μM each dNTP). The amplicons of the GPX1 gene were digested with the restriction enzyme Apa1 with 0.18 μL per sample, 0.05 μL BSA, and 1 μL 10x buffer 4, leaving fragment sizes determined by the genotype of the studied individuals (pro/pro = 191 bp; pro/leu = 191, 117, and 74 bp; leu/leu = 117 and 74 bp). Digested fragments were separated on 1% agarose and visualized by ethidium bromide staining.

In order to examine the link between groundwater selenium exposures and affective status, linear regression models were created with SPSS version 18 using GDS-30 (total and subfactor) scores as outcome variables and selenium concentration as the predictor variables. Covariates entered into the models were age, gender, education, and language of administration. In order to obtain magnitude of relations, the amount of variance accounted for by selenium concentrations alone is also presented. Logistic regression models were created to determine the risk of depression, defined as GDS score ≥ 10, that were associated with (a) groundwater selenium concentrations and (b) the GPX1 Pro198Leu polymorphism.

## Results

The mean age and education of the sample was 61.49 (sd = 12.70; range = 40-96) and 10.76 (sd = 4.37; range = 0-20), respectively. Mean GIS-based selenium concentration was 17.70 μg/L (sd = 10.66; range = 3.96-56.32). Seventy-seven percent of the sample was interviewed in English and 33% in Spanish; 88% of the sample self-reported their race as White, and 38% reported their ethnicity as Mexican American. Mean GDS-30 score of the sample was 10.49 (sd = 5.07; range = 0-26). Demographic characteristics of the sample are presented in Table 1.

**Table 1:** Demographic characteristics

	**Mean(sd)**	**Range**
Age	61.45 (12.70)	40-96
Education	10.76 (4.37)	0-20
GIS-selenium (μg/L)	17.70 (10.67)	3.96-56.32
GDS Total	10.49 (5.07)	0-26
GDS Dysphoria	3.31 (2.44)	0-11
GDS Meaninglessness	2.24 (1.43)	0-7
GDS Apathy	2.87 (1.42)	0-6
GDS Cognitive Impairment	1.87 (1.19)	0-6
GPX1 Polymorphism (%)		
CC	46	
CT	48	
TT	6	

Selenium concentrations were found to be significantly and negatively related to all GDS scores. Specifically, higher selenium levels were significantly related to lower scores in the Total GDS-30 scores (β = -0.34, p < 0.001), explaining 11% of the variance. Higher selenium was also related to a decrease in factor scores of Dysphoria (β = -0.23, p < 0.001), explaining 5% of the variance, Meaninglessness (β = -0.42, p < 0.001), explaining 17% of the variance, and Apathy (β = -0.30, p < 0.001), explaining 9% of the variance. Higher selenium concentration was statistically a significant predictor of lower scores on the subfactor of Cognitive Impairment (β = -0.11, p = 0.01), but only accounted for 1% of the variance. The results of the regression analysis can be found in Table 2. Higher selenium concentration was also associated with a significantly lower risk of depression, with B = -0.06 (SE = 0.01), Wald (df = 1) = 37.25, p < 0.001, and OR = 0.94 (95% CI = 0.92-0.96).

**Table 2:** Linear regression results

	**B(SE)**	**t**-**score**	**p**-**value**	**% Variance accounted For**
GDS Total	−0.16 (0.02)	−8.21	0.000	11%
GDS Dysphoria	−0.05 (0.01)	−5.24	0.000	5%
GDS Meaninglessness	−0.06 (0.006)	−10.31	0.000	17%
GDS Apathy	−0.04 (0.006)	−7.11	0.000	9%
GDS Cognitive Impairment	−0.13 (0.005)	−2.53	0.012	1%

The link between depression and GPX1 polymorphisms was strongest for CC (homozygous wild type) and TT (homozygous mutant type) carriers, with the greatest impact being for TT individuals as shown in Table 3. The TT polymorphism was the least frequent in the sample; however, for TT individuals, selenium concentrations accounted for 26% of the variance in GDS Total Score, 13% of the variance in Dysphoria, 37% of the variance in Meaninglessness, and 19% of the variance in Apathy. For the CC polymorphism, selenium concentrations accounted for 15% of the variance in total score, 9% of the variance in Dysphoria, 22% of the variance in Meaninglessness, and 14% of the variance in Apathy. The amount of variance for CT polymorphism was relatively small.

**Table 3:** Linear Regression Results by GPX1 Allele

	**B(SE)**	**t**-**score**	**p**-**value**	**Model variance**	**Selenium variance**
CC (n = 221)					
GDS Total	−0.18 (0.03)	−6.40	<0.001	21%	15%
GDS Dysphoria	−0.05 (0.02)	−3.24	0.001	9%	4%
GDS Meaninglessness	−0.06 (0.007)	−8.04	<0.000	24%	22%
GDS Apathy	−0.05 (0.008)	−6.10	<0.000	18%	13%
GDS Cognitive Impairment	−0.02 (0.007)	−2.96	0.003	7%	5%
CT (n = 208)					
GDS Total	−0.13 (0.04)	−3.58	<0.001	10%	6%
GDS Dysphoria	−0.05 (0.02)	−2.99	0.003	9%	4%
GDS Meaninglessness	−0.05 (0.01)	−4.68	<0.001	12%	9%
GDS Apathy	−0.03 (0.01)	−2.74	0.007	3%	3%
GDS Cognitive Impairment	0.00 (0.008)	−0.05	0.96	2%	0%
TT (n = 33)					
GDS Total	−0.27 (0.07)	−3.78	<0.001	40%	26%
GDS Dysphoria	−0.08 (0.04)	−2.27	0.03	21%	13%
GDS Meaninglessness	−0.11 (0.2)	−5.11	<0.000	53%	37%
GDS Apathy	−0.06 (0.02)	−2.81	0.009	28%	19%
GDS Cognitive Impairment	−0.02 (0.3)	−0.73	0.47	3%	2%

Next, we examined the impact of the GPX1 Pro198Leu polymorphism on the findings. First, we sought to determine if homozygous carriage of the C allele was associated with depression status (GDS ≥ 10 = depressed), compared to T allele carriers (CT plus TT; TT was not analyzed separately due to sample size). Logistic regression was conducted with depressed status as the outcome variable and GPX1 polymorphism (CC versus CT) as a categorical predictor variable. Sixty-six percent of CC homozygotes were depressed compared to 57% for T allele carriers; therefore, homozygous carriage of the C allele was associated with a significantly increased risk of depression (B = 0.50, SE = 0.19, Wald (df = 1) = 6.88, p = 0.009, and OR = 1.65 (95% CI = 1.14-2.40). Next, a linear regression model was created to examine the gene-dose effect of GPX1 C versus T (CC = 2, CT = 1, TT = 0) alleles on depression scores (GDS total score). With age, gender, education, and test language entered as covariates, the increasing presence of the T allele was associated with significantly higher GDS scores (B = 0.94, SE = 0.35, t = 2.69, and p = 0.008). Lastly, we re-ran our regression analyses examining the link between selenium concentrations and GDS scores (total and subfactors) after stratification of the sample on genotype at the GPX1 Pro198Leu polymorphism. The link between selenium levels and depression was strongest among CC and TT, as compared to CT individuals. The TT polymorphism was the least frequent in the sample. Of note, in regression analysis of groundwater selenium levels on depression risk, models that included Pro198Leu genotype along with age, gender, education, and language of test administration accounted for more variance (70%) than those that did not include genotypic status (50%). For CC polymorphism, selenium concentrations alone accounted for 15% of the variance in total score, 9% of the variance in dysphoria, 22% of the variance in meaninglessness, and 14% of the variance in apathy, which was more variance accounted for than found by covariates. The amount of variance for CT polymorphism was relatively small. See Table 3.

Therefore, these findings point to a gene-environment interaction, which is supported by the gene-dose findings.

## Discussions and conclusions

In the past three decades, selenium has been found to be essential to various aspects of health [47]. It has been found to play a role in prevention of cancer and cardiovascular disease as well as in regulating mood [20]. Exposure to environmental factors, such as selenium, can also play a role in depression and anxiety states. Past research has shown that low selenium concentrations were related to depression status and other negative affective states [18,20]. In addition, studies have indicated that selenium may be useful in preventing postpartum depression [22].

In the current study, higher groundwater selenium levels were associated with lower scores on the Geriatric Depression Scale. This finding is consistent with past research indicating that low selenium status is related to increased incidence of depression and other negative affective states [18,20]. In addition to the decrease in total depression scores, higher selenium levels were specifically related to a decrease in symptoms of Dysphoria, Meaninglessness, and Apathy, indicating that selenium may have an impact on particular types of depressive manifestations. It is interesting to note that selenium concentrations were only minimally related to the cognitive impairment subscale, as we have previously shown selenium to be selectively protective of memory functioning [36]. This suggests that, despite the potential role of selenium concentrations in preserving cognitive function, depressive symptoms related to thoughts of cognitive dysfunction appear to be unrelated and may be related to the concept of insight.

This is the first study to examine residential groundwater selenium levels and depression. A myriad of factors determine the chemical make-up of groundwater in a given area, and it is important to be cognizant of the potential health impacts from such substances. Research on groundwater and its implications on human health can have profound implications in the realms of public health and policy. Additionally, results obtained by groundwater research could prove to be the impetus for the formation or reformation of population-based prevention strategies concerning groundwater safety and exposure. This study was conducted in a rural agricultural area where higher concentrations of groundwater selenium are typically found. Aligning with this, the mean selenium level in this study was 17.70 μg/L, whereas the national average is 10 μg/L [48].

The protective impact of selenium on mental health fits with its biological activity and is supported by both animal model and human research. Selenium, a main constituent of neuronal selenoproteins, may be particularly important for sustaining healthy cerebral function [47] through its key role in protection against oxidative damage [15,20,49]. Oxidative damage is a key biological mechanism in depression pathophysiology, and many anti-depressants, like fluoxetine [50] and venlafaxine [51], reduce oxidative damage. Another possible mechanism of action is through selenium’s impact on brain-derived neurotrophic factor (BDNF), which has been linked to depression and found to be decreased in selenium deficient rats [52]. Additionally, depression is also associated with lower antioxidant levels. Research has suggested that antioxidant deficiency found in depression may predispose individuals to greater inflammatory and oxidative and nitrosative responses [13], which may contribute to the protective effects of selenium against depression.

Ours is the first study to directly examine the link between the GPX1 Pro198Leu polymorphism and depressive risk as well as its impact on the selenium-depression link. Our findings point to gene-environment interaction, which is supported by the gene-dose impact. Specifically, the C allele of the GPX1 polymorphism was associated with significantly increased risk of depression in a dose-dependent manner (i.e. CC > CT > TT). Additionally, the impact of environmental selenium concentrations on depression scores was stronger for the CC than CT group. The TT group showed a substantial impact in the expected direction, but due to the small sample number of TT homozygotes, these results must be replicated. It is also important to note the differences in effect sizes for selenium on depression observed in each genotypic group. Among CC homozygotes, the majority of variance accounted for in GDS scores was from selenium concentrations with age, gender, education, and language of test administration contributing less to GDS scores. Among the TT group, the amount of variance accounted for by selenium concentrations alone was substantial; however, covariates in the model also accounted for a larger portion of the variance. Future work is needed to delve more thoroughly into this gene-environment interaction as well as the dose-response impact of the C allele of the GPX1 polymorphism on risk for depression. Additional work is also required to examine other potential genes of interest. For example, Galecki and colleagues [53] identified polymorphisms of the NOS2A gene, which are important in the nitric oxide pathways, as related to recurrent depression. Given that selenium (i.e. selenoproteins) is involved in the nitric oxide pathway as well, NOS2A and other genes relevant to pathways linked to selenium should be examined. It is likely that more sophisticated bioinformatic analyses will be necessary to disentangle these multi-level gene-(biological mechanism) – environmental exposure – depression interactions.

These results point towards possible pharmacogenetic implications of the Pro198Leu GPX1 polymorphism. Specifically, in our sample, CC homozygotes had significantly increased risk for depression; however, selenium was also most powerfully protective against depression and depressive symptoms among those particular carriers. Therefore, future research looking at the therapeutic impact of selenium on depression should examine this genotype. Additionally, previously conducted clinical trials of anti-depressant medications with stored DNA (or previously conducted genetic analyses) can determine if Pro198Leu GPX1 genotype predicts optimal treatment response. Lastly, the Pro198Leu GPX1 polymorphism may be useful for identifying those individuals most likely to benefit from anti-depressant medications in new clinical trials.

There are limitations to the current study. One is the GIS-based estimate of selenium concentration rather than direct measurement. However, our prior work, as well as that of others, has shown that GIS-based methods are valid ways for estimating environmental exposures. The current findings should be replicated using direct selenium measurement. The cross-sectional nature of the study is another limitation; however, our prior work has shown selenium to be preventative of cognitive decline prospectively in a subset of this cohort [30]. Future studies will examine the protective impact of selenium concentrations on both depression and cognitive decline as future waves become available. The current study also has several strengths. Specifically, this is the first study to examine this link in such detail (i.e. depressive symptoms rather than only global scores) as well as look at this topic among rural-dwelling adults and elders. This is also the first study to examine the impact of genetic variation within GPX1 on depression risk as well as the selenium-depression link. Taken together, these results point to the need for continued work in the area, as selenium may provide a therapeutic target for preventative strategies aimed at reducing depressive incidence rates in vulnerable populations. Additional work is also needed to determine the pharmacogenetic implications of the Pro198Leu GPX1 polymorphism in depression therapies.

## Abbreviations

CBPR: Community-based participatory research; GDS: Geriatric depression scale; GIS: Geospatial information system; GPX1: Glutathione peroxidase 1; SNP: Single nucleotide polymorphism; TWDB: Texas water development board; BDNF: Brain-derived neurotrophic factor; PCRs: Polymerase chain reactions; CC: Homozygous wild type alleles; TT: Homozygous mutant type alleles.

## Competing interests

The authors declare no competing financial interests. This work was funded in part by the Science To Achieve Results (STAR) Program of the U.S. Environmental Protection Agency (EPA) grant # RD83479401 (O’Bryant, PI), National Institute on Minority Health and Health Disparities (NIMHD) award #L60MD001849 and the Hogg Foundation grant # JRG-040 (O’Bryant, PI).

## Authors’ contributions

LJ: Designed the study, participated in data collection and analysis, and manuscript preparation. JP: Participated in data collection, entry, and manuscript preparation. CM: Participated in data collection and manuscript preparation. ME: Participated in data collection, and manuscript preparation. VB: Participated in data collection, analysis, and manuscript preparation. JH: Assisted in study design, data analysis, and manuscript preparation. RB: Assisted in study design, data analysis, and manuscript preparation. TC: Assisted in data analysis, and manuscript preparation. EH: Participated in manuscript preparation. SO: Designed the study, participated in data collection and analyses, and manuscript preparation. All authors have read and approved this manuscript.

## Pre-publication history

The pre-publication history for this paper can be accessed here:

http://www.biomedcentral.com/1471-244X/13/7/prepub
